# Prevalence of dietary supplement use and associated factors among female college students in Saudi Arabia

**DOI:** 10.1186/s12905-017-0475-y

**Published:** 2017-11-22

**Authors:** Hanan Alfawaz, Nasiruddin Khan, Aziza Alfaifi, Fatima M. Shahrani, Huda M. Al Tameem, Seetah F. Al Otaibi, Weaam I. Abudigin, Mohammad S. Al-Shayaa, Saad A. Al-Ghanim, Nasser M. Al-Daghri

**Affiliations:** 1 College of Food Science & Agriculture, Department of Food Science & Nutrition, King Saud University, Riyadh, Saudi Arabia; 2 Prince Mutaib Chair for Biomarkers of Osteoporosis, Biochemistry Department, King Saud University, Riyadh, 11451 Saudi Arabia; 3 College of Applied Sciences, Department of Food Science and Human Nutrition, A’Sharqiyah University, Ibra, 400 Sultanate of Oman; 4 Department of Agricultural Extension and Rural Sociology, College of Foods and Agricultural Sciences, King Saud University, Riyadh, Saudi Arabia; 5 Health and Hospital Administration Program, Department of Health Administration, College of Business Administration, King Saud University, Riyadh, Saudi Arabia

**Keywords:** Dietary supplements, Sociodemographic, Lifestyle, Saudi Arabia

## Abstract

**Background:**

The economic boom in Saudi Arabia indirectly prompted the use of dietary supplements in the last two decades. Our aim is to investigate the prevalence of dietary supplement use and its association with sociodemographic/lifestyle characteristics among Saudi female students.

**Methods:**

In this cross-sectional study, 534 female participants (≥19 years of age) completed a self-administered questionnaire that include sociodemographic and lifestyle characteristics, perceived health status, dietary supplement use, general awareness, attitudes and behavior.

**Results:**

In all participants, the prevalence of dietary supplement use was 76.6% (*n* = 409). High level of education (*p* = 0.002) and more physical activity (*p* = 0.008) exhibited a significant positive association with users than to non-users. The frequency showed that beta-carotene (54.2%), chamomile (54.2%), and glucosamine (53.8%) were the most preferred diet supplements under the category “when needed”. Cod liver oil (71.3%), omega 3 (68.3%), multi-vitamins (61.5%), ginseng (60%), and vitamin A (60%), were mostly used “from time to time”. Multi-minerals (34.4%) were the preferred choice when it comes to daily use. The main reasons for supplement use were to “maintain healthy hair” and “injury and illness” (both 26.2%). About 38.4% were not aware and 30.3% disagree on differences taking supplements with or without consulting a medical professional. About 36.7% lack information about side effects while, 35.0% were unaware about any health effect of dietary supplements.

**Conclusion:**

The prevalence of dietary supplement use was high in Saudi female students and was significantly associated with sociodemographic and lifestyle factors.

## Background

The Kingdom of Saudi Arabia is a fast growing economic country that has affected its general population in various ways including a transition in daily lifestyle patterns and dietary intake habits. The pace of change has diverted the traditional Saudi diet towards the so-called Western diets, with increased consumption of energy dense and processed foods leading towards increased prevalence of non-communicable diseases in many Arab regions [1, 2].

Studies from several regions in Saudi Arabia in different age groups and sex have shown overweight- and obesity-related demographic, socioeconomic status and physical activity factors [3–6]. A recent study performed by our group in Saudi children has shown the influence of knowledge and attitude on vitamin D status [7]. In Saudi Arabia, 66% of adult men and 71% of adult women are either overweight or obese [8], indirectly contributing to a very high healthcare burden [9]. Emerging scientific evidence regarding the positive and negative effects of dietary supplements has opened more options to maintain a healthy life [10].

Dietary supplements are food products containing dietary ingredients intended to add more nutritional value to a normal diet [11]. There is a lot of evidence on the widespread use of dietary supplements in US and Europe [12–18]. In GCC countries, reports have demonstrated an increase in demand for nutritional supplements [19–22]. For instance, a recent study performed among college students in Qatar revealed a high percentage (49.6%) use of nutritional and herbal supplements. In addition, the use of complementary and alternative medicines in the general population and patients with diabetes have been observed in Bahrain and Saudi Arabia, respectively [20, 23]. There are studies regarding the use and awareness of dietary/multi-vitamin and folic acid supplements in pregnant Saudi women [24] as well as vitamin D supplements in Saudi female outpatients [25]. However, only one study exhibited the usage pattern of dietary supplements in professional Saudi male athletes [26]. There is no study present till date providing information about the determinants of dietary supplement use in the female population of Saudi Arabia. To the best of our knowledge, this is the first study of its kind to examine the use of dietary supplements and it association with sociodemographic/lifestyle factors, behavior, and awareness among Saudi female students.

## Methods

### Sample size calculation

Sample size calculation was based on existing literature [27]. With the prevalence of 39% of dietary supplement use in college students, 5% margin of error, the required sample size at 95% confidence interval is 366 patients. After adjusting for non-response of 30%, 534 female participants were enrolled in the study.

### Study population

The sample population of this cross-sectional study included 319 students randomly selected from science and health colleges (Medicine, Science, Pharmacy, Information Technology, Dentistry and Applied Medical Science) and 215 from Humanitarian colleges (Education, Business Administration, Arts, Tourism/Antique, Law/Political Science and Language/Translation) of King Saud University, Riyadh, Saudi Arabia. All 534 participants were females aged 19 to 26 years.

### Data collection and measurements

A pilot study (10 students) was performed to confirm the reliability and validity of the questionnaire by using different approaches. We distributed the questionnaire amongst students to get their feedback regarding the understanding and clarity of all questions. The questionnaire was then reviewed by experts in related fields as well as other expert colleagues within the university. We also asked external reviewers to provide their feedback and opinion in developing/improving the questionnaire to ensure reliability of the test and compared the results of our pilot study with the results of similar work done previously. We introduced all necessary expert feedback and suggestions accordingly until we had a final questionnaire which was used in the present study.

The participants were asked to complete the self-administered questionnaire. It was divided into four parts including sociodemographic/lifestyle characteristic, frequency, type and reason of supplement used in different circumstances and awareness and attitude about use of dietary supplements. Income <5000 Saudi Arabia riyals (SAR) was considered low, between 5000 and 9999 SAR was considered average, 10,000–16,000 SAR was considered moderate and >16,000 SAR was considered high. The type of physical activity included walking, resistance exercises, swimming and dance. The questionnaire also included sources of spending, motivations, and circumstances for use of dietary supplements. Participants were also asked to respond for the importance of physician’s role in diet supplement purchases.

Regarding dietary supplement use, participants were asked whether they were currently taking any supplement together about information on the frequency and duration of use for each dietary supplement reported.

### Data analysis

Cronbach’s α, an estimate of coefficient of reliability, was measured for the questionnaire and the value obtained was 84% (excellent). The association between sociodemographic/lifestyle characteristics of respondents and use of supplements were assessed using the Chi-square test or Fisher exact test. Type and use of dietary supplements, reason for use, awareness and attitude, sources of information, spending and purchases were presented as frequency distributions. Analyses were performed using SPSS software, version 16 (SPSS, Chicago, IL, USA). The significance level was set at *P* < 0.05.

## Results

### Sociodemographic and lifestyle characteristics

Table 1 represents the sociodemographic/lifestyle characteristics of the participants (*n* = 534) (age range; 19–21 years), 23.4% females (age range; 22–24 years), 9.7% females (age range; 24–26 years), and 14.4% females (age range; >27 years). The majority of the respondents showed normal BMI (60.9%), marital status (single, 74.5%), higher family income (16,000 SAR, 31.8%), and education level (3rd – 5th level, 44.2%).

**Table 1:** Participants Sociodemographic/lifestyle Characteristics

Demographics	N (%)
Age (years)	
19–22	280 (52.4)
22–24	125 (23.4)
24–26	52 (9.7)
> 26	77 (14.4)
BMI Status (kg/m^2^)	
Normal	325 (60.9)
Overweight	148 (27.7)
Obese	61 (11.4)
Social Status	
Married	136 (25.5)
Single	398 (74.5)
Monthly Family Income (SAR)	
< 5000	57 (10.7)
5000–10,000	159 (29.8)
10,000–16,000	148 (27.7)
> 16,000	170 (31.8)
The Academic Track	
Scientific and Medical Colleges	319 (59.7)
Humanity Colleges	215 (40.3)
Educational Level:	
3rd – 5th Level	236 (44.2)
6th – 8th Level	201 (37.6)
> 8th Level	97 (18.2)

Note: Data presented as frequencies (%)

Table 2 shows the prevalence of dietary supplement use, distribution of participant’s dietary history and the type of physical activity. The prevalence of dietary supplement use was 76.6% (*n* = 409) among female students. In the study population 92.7% were nonsmokers and 74% responded positive for daily physical activity with walking as most preferred type.

**Table 2:** Participants History of Disease, Physical activity, Prevalence of Dietary Supplements

Parameters	Dietary Supplement Use		*P*-values
	Yes	No	
Age (in Years)			0.234
19–22	206 (73.6)	74 (26.4)	
22–24	97 (77.6)	28 (22.4)	
24–26	41 (78.8)	11 (21.2)	
Higher than 26	65 (84.4)	12 (15.6)	
Family Income (in Saudi Riyals)			0.135
Less than 5000	40 (70.2)	17 (29.8)	
5000–10,000	131 (82.4)	28 (17.6)	
10,000–16,000	114 (77.0)	34 (23.0)	
Higher than 16,000	124 (72.9)	46 (27.1)	
Marital Status			0.171
Married	110 (80.9)	26 (19.1)	
Single	299 (75.1)	99 (24.9)	
BMI Status			0.227
Normal	244 (75.1)	81 (24.9)	
Overweight	113 (76.4)	35 (23.6)	
Obese	52 (85.2)	9 (14.8)	
The Academic Track			0.164
Scientific and Medical	251 (78.7)	68 (21.3)	
Humanities	158 (73.5)	57 (26.5)	
Education Level			0.002
3rd – 5th Level	167 (70.8)	69 (29.2)	
6th – 8th Level	156 (77.6)	45 (22.4)	
> 8th Level	86 (88.7)	11 (11.3)	
Health Problems			0.095
Vitamin D deficiency	76 (90.5)	8 (9.5)	
Other Disease	76 (81.7)	17 (18.3)	
Physical Activity			0.008
Physical Activity (Yes)	314 (79.5)	81 (20.5)	
Resistance Exercise + Walking (Yes)	256 (79.5)	66 (20.5)	0.050
Walk (Yes)	211 (79.0)	56 (21.0)	0.184
Resistance Exercise (Yes)	45 (81.8)	10 (18.2)	0.334
Swimming (Yes)	41 (78.8)	11 (21.2)	0.686
Dance (Yes)	29 (80.6)	7 (19.4)	0.561

Note: Data presented as frequencies (%)

The association between use of dietary supplements and socioeconomic, demographic factors and physical activity are presented in Table 3. A high level of education (*P* = 0.002), and more physical activity (*P* = 0.008) were both significantly associated with use of dietary supplement than non-users.

**Table 3:** Association between Use of Dietary Supplements, Age, Marital status, Family Income, BMI, Academic Track, Educational Level and Health Problems, physical activity

Parameters	Dietary Supplement Use		*P*-values
	Yes	No	
Age (in Years)			0.234
19–22	206 (73.6)	74 (26.4)	
22–24	97 (77.6)	28 (22.4)	
24–26	41 (78.8)	11 (21.2)	
Higher than 26	65 (84.4)	12 (15.6)	
Family Income (in Saudi Riyals)			0.135
Less than 5000	40 (70.2)	17 (29.8)	
5000–10,000	131 (82.4)	28 (17.6)	
10,000–16,000	114 (77.0)	34 (23.0)	
Higher than 16,000	124 (72.9)	46 (27.1)	
Marital Status			0.171
Married	110 (80.9)	26 (19.1)	
Single	299 (75.1)	99 (24.9)	
BMI Status			0.227
Normal	244 (75.1)	81 (24.9)	
Overweight	113 (76.4)	35 (23.6)	
Obese	52 (85.2)	9 (14.8)	
The Academic Track			0.164
Scientific and Medical	251 (78.7)	68 (21.3)	
Humanities	158 (73.5)	57 (26.5)	
Education Level			0.002
3rd – 5th Level	167 (70.8)	69 (29.2)	
6th – 8th Level	156 (77.6)	45 (22.4)	
> 8th Level	86 (88.7)	11 (11.3)	
Health Problems			0.095
Vitamin D deficiency	76 (90.5)	8 (9.5)	
Other Disease	76 (81.7)	17 (18.3)	
Physical Activity			0.008
Physical Activity (Yes)	314 (79.5)	81 (20.5)	

Note: Data presented as frequencies (%). *P* < 0.05

### Types and use of dietary supplement

Table 4 shows different types of dietary supplements consumed by the participants at different circumstances: vitamins (A, D, and C), beta-carotene, vitamin B group, vitamin B12, multivitamins, minerals, vitamin/mineral complexes, muti-minerals ginseng, *Ginkgo biloba*, chamomile, garlic capsules, CO enzyme, active protein, omega-3, cod liver oil, and glucosamine.

**Table 4:** Types and Use of Dietary Supplements

Supplements	As needed	From time to time	Daily	When Sick
Active Protein	9 (42.9)	6 (28.6)	5 (23.8)	1 (4.8)
Beta-carotene	13 (54.2)	7 (29.2)	4 (16.7)	0
Calcium	30 (28.3)	59 (55.7)	8 (7.5)	9 (8.5)
Chamomile	13 (54.2)	11 (45.8)	0	0
Chromium	7 (36.8)	6 (31.6)	4 (21.1)	2 (10.5)
CO enzyme	8 (47.1)	6 (35.3)	2 (11.8)	1 (5.9)
Cod liver oil	15 (18.8)	57 (71.3)	7 (8.8)	1 (1.3)
Folic acid	30 (34.1)	43 (48.9)	9 (10.2)	6 (6.8)
Garlic capsules	11 (42.3)	14 (53.8)	0	1 (3.8)
*Ginkgo biloba*	7 (33.3)	12 (57.1)	0	2 (9.5)
Ginseng	6 (30.0)	12 (60.0)	0	2 (10.0)
Glucosamine	7 (53.8)	4 (30.8)	1 (7.7)	1 (7.7)
Iron	40 (27.6)	66 (45.5)	16 (11.0)	23 (15.9)
Magnesium	8 (33.3)	11 (45.8)	3 (12.5)	2 (8.3)
Multi minerals	8 (25.0)	13 (40.6)	11 (34.4)	0
Multivitamins	19 (19.8)	59 (61.5)	14 (14.6)	4 (4.2)
Omega-3	14 (17.1)	56 (68.3)	11 (13.4)	1 (1.2)
Potassium	7 (28.0)	11 (44.0)	4 (16.0)	3 (12.0)
Vitamin A	18 (22.5)	48 (60.0)	11 (13.8)	3 (3.8)
Vitamin B12	20 (30.8)	37 (56.9)	6 (9.2)	2 (3.1)
Vitamin B Group	22 (30.6)	42 (58.3)	6 (8.3)	2 (2.8)
Vitamin C	16 (25.8)	34 (54.8)	6 (9.7)	6 (9.7)
Vitamin D	55 (27.1)	114 (56.2)	13 (6.4)	21 (10.3)
Zinc	12 (20.7)	36 (62.1)	7 (12.1)	3 (5.2)

Note: Data presented as frequencies (%)

409 (76.6%) students used at least one of the above mentioned dietary supplements in the past 12 months preceding the study. Based on four categories (when it is needed, from time to time, daily, and during disease), the frequency for dietary supplements use showed that beta-carotene (54.2%), chamomile (54.2%), and glucosamine (53.8%) were the most preferred diet supplements in the category “when needed”. Cod liver oil (71.3%), omega 3 (68.3%), multi-vitamins (61.5%), ginseng (60%), and vitamin A (60%), were among the most frequently used supplement under the category (from time to time). Multi-minerals (34.4%) was the preferred choice for daily use. Iron (15.9%) and potassium (12%) supplements were common in the diseased category (Table 4).

### Reasons for the use of dietary supplements

**Fig. 1:**
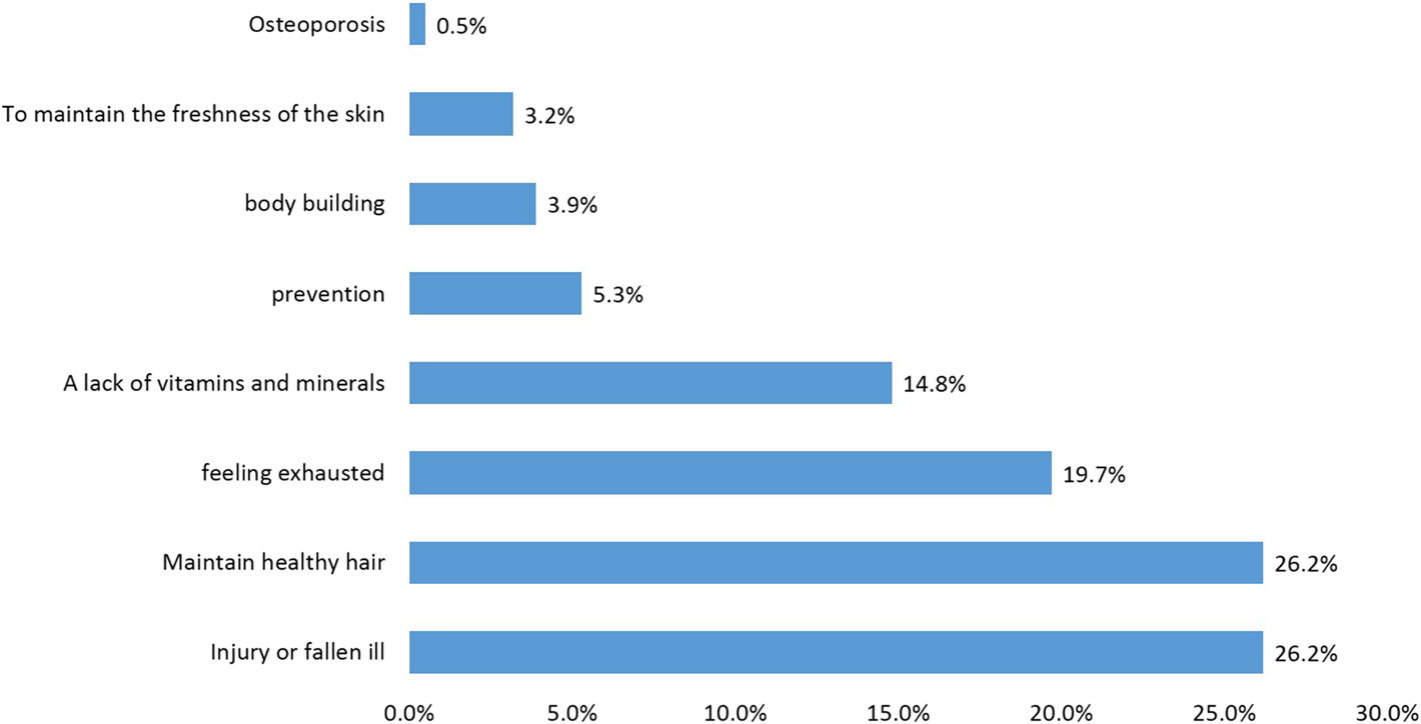
Reasons for using Dietary Supplements

Reasons for the use of dietary supplements was presented in Fig. 1. These include to “maintain healthy hair” and in “injury and illness” (both at 26.2%).

### Sources of spending, purchase information, and duration of use of dietary supplementations

The sources of spending, motivation, circumstances, duration of use and sources of information about dietary supplementation are presented in Table 5. Dietary supplements were mainly purchased at their own expense (76.7%) following only a medical prescription (79.3%) for a maximum of two weeks (23.9%). Social media was the most common source (38%) of information for dietary supplements.

**Table 5:** Source of Spending, Purchase, Duration of Use and Sources of Information dietary supplementations

Survey Questions	N (%)
What is the source of spending on food supplements?	
On your account	339 (76.7)
Free	61 (13.8)
Insurance	42 (9.5)
The purchase of nutritional supplements by?	
Prescription	329 (79.3)
Pharmacies	63 (15.2)
Internet	22 (5.3)
The private	1 (0.2)
What is the duration of your use of nutritional supplements?	
2 weeks	104 (23.9)
From time to time	97 (22.2)
A month	91 (20.9)
Daily	62 (14.2)
Weekly	29 (6.7)
3 Months	25 (5.7)
6 Months	15 (3.4)
One year	13 (3.0)
Information Sources on Supplements	
Social Media	203 (38.0)
Internet	85 (15.9)
Newspapers	58 (10.9)
Family	44 (8.2)
More than one source	40 (7.5)
Physician	39 (7.3)
Pharmacies	28 (5.2)
Friends	26 (4.9)
The books	11 (2.1)

Note: Data presented as frequencies (%)

### Awareness and attitude about supplements use

Table 6 lists the questions about the general awareness and attitudes in the use of dietary supplements. Majority of the participants (70.6%) use dietary supplements following a doctor’s prescription but 38.4% do not know the difference between taking the supplements with or without consulting a medical professional while 30.3% claim there is no difference. Majority of them (40.1%) read the attached guide/medical instructions before use of dietary supplements. About 36.9% agreed that there are negative side effects of dietary supplements with 36.7% of students unaware of this fact. Almost 40% of the participants responded positively to the consideration of food supplements being essential for their health. However, 35% were unaware of this information.

**Table 6:** Awareness and Attitudes about Supplement Use

Awareness and Attitudes Do you...	Yes	No	Sometimes	I don’t know
Take dietary supplements based on a prescription through a doctor?	377 (70.6)	55 (10.3)	56 (10.5)	46 (8.6)
Think that there is a difference between taking the supplements with or without by medical consult?	115 (21.5)	162 (30.3)	52 (9.7)	205 (38.4)
Read the attached instructions with the supplement?	214 (40.1)	142 (26.6)	61 (11.4)	117 (21.9)
Think that dietary supplements have any negative side effects?	197 (36.9)	74 (13.9)	67 (12.5)	196 (36.7)
See the food supplements essential for your health?	212 (39.7)	38 (7.1)	97 (18.2)	187 (35.0)
Think that dietary supplements substitute for food diversity	65 (12.2)	205 (38.4)	54 (10.1)	210 (39.3)
Know that you have to do lab test to check levels of vitamins and minerals?	194 (36.3)	252 (47.2)	88 (16.5)	–

Note: Data presented as frequencies (%)

## Discussion

The present study demonstrated the high prevalence of dietary supplement use and its association with sociodemographic and lifestyle factors in female student at King Saud University, Saudi Arabia. The association between higher education level and dietary supplement use has been shown in various studies. Pouchieu and colleagues demonstrated a significant direct association of higher level of education and dietary supplement use in French adult population [28]. Similarly, a study performed by Mileva-Peceva et al. reported a significantly higher consumption of vitamins and/or mineral food supplements in females with a higher educational status [18]. Our present study supports the above findings showing a significant direct association between level of education and dietary supplements use.

Studies across different populations and sex show a healthier lifestyle associated with dietary supplement use [29–31]. A study performed by Kim et al. [32] in a Korean population (men and women) showed that dietary supplement users were more likely to be engaged in moderate or vigorous physical activity. Pouchieu and colleagues [28] also reported that women with higher use of dietary supplements showed high level of physical activity. Our present study supported the above results showing a significant direct association of physical activity with dietary supplement users. Although awareness of dietary supplements use based on a prescription by a physician was high (70.6%, and 79.3%, respectively), participants lack proper information and basic knowledge about side effects, importance of doctor’s prescription and reliable source. A possible cause for this unawareness could be the lack of proper counseling and recommendations about healthy diet from time to time via reliable sources like physicians and experts. The present study has some limitations and should be considered before extrapolating the results to the general public. The present findings cannot be generalized due to small sample size, which is not representative of the overall female population in Saudi Arabia. Due to the cross-sectional design of this study, the reported associations, particularly with respect to sociodemographic/lifestyle characteristic and health outcomes could not establish causality.

## Conclusions

In conclusion, the present study provided new information regarding the high prevalence of dietary supplement use among females in Saudi Arabia. The study reported a significant direct association between higher level of education, physical activity and the use of dietary supplements. Moreover, the study emphasizes the need for increased awareness and basic knowledge related to side effects and source of reliable information for the use of dietary supplements. Finally, the present study highlights the need to have expert healthcare practitioners in the related field for proper and timely guidance in general population.

## Abbreviations

BMI: Body mass index
GCC: Gulf Cooperation Council
MOH: Ministry of Health
SAR: Saudi Arabian Riyals
SPSS: Statistical Package for the Social Sciences
UAE: United Arab Emirates

## References

[1] Al-Hazzaa HM (2002). Physical activity, fitness and fatness among Saudi children and adolescents: implications for cardiovascular health. Saudi Med J.

[2] Musaiger AO (2004). Overweight and obesity in the eastern Mediterranean region: can we control it?. East Mediterr Health J.

[3] Washi SA, Ageib MB (2010). Poor diet quality and food habits are related to impaired nutritional status in 13- to 18-year-old adolescents in Jeddah. Nutr Res.

[4] Al-Daghri NM, Al-Attas OS, Alokail MS, Alkharfy KM, Yakout SM, Sabico SB, Gibson GC, Chrousos GP, Kumar S (2011). Parent-offspring transmission of adipocytokine levels and their associations with metabolic traits. PLoS One.

[5] Al-Nuaim AA, Al-Nakeeb Y, Lyons M, Al-Hazzaa HM, Nevill A, Collins P, Duncan MJ (2012). The prevalence of physical activity and sedentary behaviours relative to obesity among adolescents from Al-Ahsa, Saudi Arabia: rural versus urban variations. J Nutr Metab..

[6] Al-Daghri NM, Al-Othman A, Alkharfy KM, Alokail MS, Khan N, Alfawaz HA, Aiswaidan IA, Chrousos GP (2012). Assessment of selected nutrients intake and adipocytokines profile among Saudi children and adults. Endocr J.

[7] Al-Daghri NM, Alkharfy KM, Al-Attas OS, Khan N, Alfawaz HA, Alghanim SA, Al-Yousef MA, Al-Ajlan AS, Alokail MS (2014). Gender-dependent associations between socioeconomic status and metabolic syndrome: a cross-sectional study in the adult Saudi population. BMC Cardiovasc Disord.

[8] Ng SW, Zaghloul S, Ali HI, Harrison G, Popkin BM (2011). The prevalence and trends of overweight, obesity and nutrition-related non-communicable diseases in the Arabian gulf states. Obes Rev.

[9] Diabetes Atlas, International diabetes federation, 2010. http://www.diabetesatlas.com/content/middle-east-and-northafrica (Last accessed on 08 October 2016).

[10] Ernst E, Pittler MH, Wider B (2006). The desktop guide to complementary and alternative medicine.

[11] DSHEA, Dietary Supplement Health and Education Act of 1994. Pub L No103–417, 108 Stat 4325, 994.

[12] Bailey RL, Gahche JJ, Lentino CV, Dwyer JT, Engel JS, Thomas PR, Betz JM, Sempos CT, Picciano MF (2011). Dietary supplement use in the United States, 2003–2006. J Nutr.

[13] Gahche J, Bailey R, Burt V, Hughes J, Yetley E, Dwyer J, Picciano MF, McDowell M, Sempos C (2011). Dietary supplement use among U.S. adults has increased since NHANES III (1988–1994). NCHS Data Brief.

[14] Gardiner P, Kemper KJ, Legedza A, Phillips RS (2007). Factors associated with herb and dietary supplement use by young adults in the United States. BMC Complement Altern Med.

[15] Murphy SP, Wilkens LR, Monroe KR, Steffen AD, Yonemori KM, Morimoto Y, Albright CL (2011). Dietary supplement use within a multiethnic population as measured by a unique inventory method. J Am Diet Assoc.

[16] Skeie G, Braaten T, Hjartåker A, Lentjes M, Amiano P, Jakszyn P, Pala V, Palanca A, Niekerk EM, Verhagen H, Avloniti K, Psaltopoulou T, Niravong M, Touvier M, Nimptsch K, Haubrock J, Walker L, Spencer EA, Roswall N, Olsen A, Wallström P, Nilsson S, Casagrande C, Deharveng G, Hellström V, Boutron-Ruault MC, Tjønneland A, Joensen AM, Clavel-Chapelon F, Trichopoulou A, Martinez C, Rodríguez L, Frasca G, Sacerdote C, Peeters PH, Linseisen J, Schienkiewitz A, Welch AA, Manjer J, Ferrari P, Riboli E, Bingham S, Engeset D, Lund E, Slimani N (2009). Use of dietary supplements in the European prospective investigation into cancer and nutrition calibration study. Eur J Clin Nutr.

[17] Marques-Vidal Pl, Pécoud A, Hayoz D, Paccaud F, Mooser V, Waeber G, Vollenweider P (2009). Prevalence and characteristics of vitamin or dietary supplement users in Lausanne, Switzerland: the CoLaus study. Eur J Clin Nutr.

[18] Mileva-Peceva Rl, Zafirova-Ivanovska B, Milev M, Bogdanovska A, Pawlak R (2011). Sociodemographic predictors and reasons for vitamin and/or mineral food supplement use in a group of outpatients in Skopje. Prilozi.

[19] GCC pharmaceutical industry report 2013. Dubai, United Arab Emirates: Alpen Capital; 2013 (http://www.alpencapital.%20com/media-reports-2013.htm, Last accessed 18 July 2014).

[20] Al-Faris EA, Al-Rowais N, Mohamed AG, Al-Rukban MO, Al- Kurdi A (2008). Balla al-Noor MA, al-Harby S, sheikh a. Prevalence and pattern of alternative medicine use: the results of a household survey. Ann Saudi Med.

[21] Al Shaar IA, Ismail MF, Yousuf WA, Salama RE (2010). Knowledge, attitudes and practice of general practitioners towards complementary and alternative medicine in Doha. Qatar East Mediterr Health J.

[22] Mathew El, Muttappallymyalil J, Sreedharan J, Lj J, John J, Mehboob M, Mathew A (2013). Self-reported use of complementary and alternative medicine among the health care consumers at a tertiary care center in Ajman, United Arab Emirates. Ann Med Health Sci Res.

[23] Khalaf AJ, Whitford DL. The use of complementary and alternative medicine by patients with diabetes mellitus in Bahrain: a cross-sectional study. BMC Complement Altern Med. 2010;14;10:35. 10.1186/1472-6882-10-35 PMC2912778 20630070

[24] Nora A. Al-Faris. High Prevalence of Vitamin D Deficiency among Pregnant Saudi Women. Nutrients. 2016;4;8(2):77. 10.3390/nu8020077 PMC4772041 26861386

[25] Kanan RMl, Al Saleh YM, Fakhoury HM, Adham M, Aljaser S, Tamimi W (2013). Year-round vitamin D deficiency among Saudi female out-patients. Public Health Nutr.

[26] Sulaiman O (2013). Aljaloud and Salam a. Ibrahim. Use of dietary supplements among professional athletes in Saudi Arabia. J Nutr Metab.

[27] Alhomoud FK, Basil M, Bondarev A (2016). Knowledge, attitudes and practices (KAP) relating to dietary supplements among health sciences and non-health sciences students in one of the universities of United Arab Emirates (UAE). J Clin Diagn Res.

[28] Pouchieu Cl, Andreeva VA, Péneau S, Kesse-Guyot E, Lassale C, Hercberg S, Touvier M (2013). Sociodemographic, lifestyle and dietary correlates of dietary supplement use in a large sample of French adults: results from the NutriNet-Santé cohort study. Br J Nutr.

[29] Marques-Vidal P, Arveiler D, Evans A, Montaye M, Ruidavets JB, Haas B (2000). Characteristics of male vitamin supplement users aged 50–59 years in France and Northern Ireland: the PRIME study. Prospective epidemiological study of myocardial infarction. Int J Vitam Nutr Res.

[30] Block G, Jensen CD, Norkus EP, Dalvi TB, Wong LG, McManus JF (2007). Usage patterns, health, and nutritional status of long-term multiple dietary supplement users: a cross sectional study. Nutr J.

[31] Radimer KL, Subar AF, Thompson FE (2000). Nonvitamin, nonmineral dietary supplements: issues and findings from NHANES III. J Am Diet Assoc.

[32] Kim Jl, Lee JS, Shin A, Kang MH, Shin DS, Chung HR, Kim WK (2010). Socio-demographic and lifestyle factors are associated with the use of dietary supplements in a Korean population. J Epidemiol.

